# External Fixation for War-Related Mandibular Fractures in a Resource-Limited Setting: A Retrospective Study of 91 Patients

**DOI:** 10.3390/jcm15020736

**Published:** 2026-01-16

**Authors:** Franck Masumbuko Mukamba, Liévin Muhindo, Marie-Hélène Bisimwa, Paul Budema, Fabrice Cikomola, Georges Kuyigwa, Olivier Cornu, Gregory Reychler, Hervé Reychler, Raphael Olszewski

**Affiliations:** 1 Surgery Department, Hôpital Provincial Général de Référence de Bukavu, Université Catholique de Bukavu, Bukavu 285, Congo; lievin.muhindo@gmail.com (L.M.); igegabisimwa13@gmail.com (M.-H.B.); paulbdma@yahoo.fr (P.B.); cikomolafab@gmail.com (F.C.); kuyigwa2002@yahoo.fr (G.K.); 2 Oral and Maxillofacial Surgery Lab (OMFS Lab), Neuro Musculo Skeletal Lab (NMSK), Institut de Recherche Expérimentale et Clinique (IREC), Université Catholique de Louvain (UCLouvain), 1200 Brussels, Belgium; raphael.olszewski@saintluc.uclouvain.be; 3 Division of Orthopedics and Musculoskeletal Trauma, Cliniques Universitaires Saint Luc, Université Catholique de Louvain (UCLouvain), 1200 Brussels, Belgium; olivier.cornu@saintluc.uclouvain.be; 4 Neuro Musculo Skeletal Lab (NMSK), Institut de Recherche Expérimentale et Clinique (IREC), Université Catholique de Louvain (UCLouvain), 1200 Brussels, Belgium; 5 Service de Kinésithérapie et Ergothérapie, Institut de Recherche Expérimentale et Clinique (IREC), Cliniques Universitaires Saint Luc, Université Catholique de Louvain (UCLouvain), 1200 Brussels, Belgium; gregory.reychler@saintluc.uclouvain.be; 6 Department of Oral and Maxillofacial Surgery, Cliniques Universitaires Saint Luc, Université Catholique de Louvain, 1200 Brussels, Belgium; reychlerherve@gmail.com; 7 Department of Perioperative Dentistry, L. Rydygiera Collegium Medicum, Nicolaus Copernicus University, 85-067 Bydgoszcz, Poland

**Keywords:** war-related injury, external fixation, mandibular fracture, outcome and complications

## Abstract

**Background/Objectives**: War-related mandibular injuries result in extensive soft-tissue damage, severe comminution, and bone loss, and are associated with high rates of infection and delayed healing. No universally accepted management protocol exists for these injuries. External fixation is commonly used in this context, particularly when internal fixation is unavailable or contraindicated. This study aimed to analyze injury patterns, treatment outcomes, and complications of war-related mandibular fractures treated with external fixation as a primary and definitive stabilization method in a resource-limited setting in eastern Democratic Republic of Congo. **Methods**: A retrospective review was conducted of all patients who sustained war-related mandibular fractures and were treated with external fixation between January 2017 and December 2024 at the Hôpital Provincial Général de Référence de Bukavu. Demographic data, injury characteristics, treatment details, outcomes, and complications were collected. Factors associated with delayed union and fracture-related infection were evaluated using univariate analysis. **Results**: Ninety-one patients with severe mandibular war injuries were included. High-velocity gunshot wounds accounted for 94.5% of injuries. Clinical evidence of wound infection at admission was present in 29.7% of patients. The mean delay between injury and external fixation was 9.2 ± 6.6 days. Successful bone healing without secondary bone procedures was achieved in 71 patients (78.0%), with a mean healing time of 7.6 ± 3.0 weeks. Delayed bone grafting was required in 20 patients (22.0%), performed at a mean of 77.3 ± 30.5 days after initial fixation. The overall complication rate was 36.3%, with fracture-site infection being the most frequent complication (30.8%). Bone loss at presentation, clinical infection at admission, and the need for bone grafting were significantly associated with fracture-related infection (*p* < 0.05). **Conclusions**: War-related mandibular fractures in this series were characterized by severe comminution, bone loss, infection, and delayed presentation. Despite these challenges, external fixation provided acceptable fracture healing and functional outcomes. Small orthopedic external fixators represent a pragmatic and effective treatment option for complex mandibular war injuries in resource-limited settings.

## 1. Introduction

For several decades, armed conflict has severely affected the eastern part of the Democratic Republic of Congo (DRC). As a consequence, war-related firearm and blast injuries have become increasingly frequent, with maxillofacial structures frequently involved [1,2]. The use of high-caliber military weapons in these conflicts results in devastating injuries characterized by extensive soft tissue damage, severe comminution, bone loss, and a high risk of contamination and infection [3,4,5]. Mandibular injuries may result from rifle bullets, missiles, grenades, bombs, or other explosive devices.

Despite the severity and complexity of these injuries, there is currently no universally accepted treatment protocol for high-velocity war-related mandibular fractures [6]. In contrast, low-velocity mandibular fractures, which are more commonly encountered in civilian practice in Western countries and the United States (US), are generally less destructive and are most often managed with open reduction and internal fixation (ORIF) using miniplate osteosynthesis [7,8]. However, in the setting of high-energy ballistic injuries with extensive contamination and devitalized bone, internal fixation may be associated with a high rate of complications, including sequestration of bone fragments, avascular necrosis due to periosteal stripping, wound infection, and osteomyelitis [9].

External fixation (EF) and maxillomandibular fixation (MMF) have traditionally been considered treatment options for highly comminuted mandibular fractures [10,11,12,13]. MMF alone is frequently inadequate in war-related injuries because dentition is often absent or severely compromised as a result of the high-energy projectile [14]. EF offers several advantages in this context, including stable fracture immobilization, minimal additional insult to soft tissues, preservation of local vascularity, unrestricted access for wound care, and maintenance of mouth opening for oral hygiene and nutrition [15]. EF may be used either as a temporary stabilization method or as a definitive treatment, while also providing an effective stage for delayed reconstruction when required [16,17,18].

In recent military conflicts in Iraq and Afghanistan, temporary EF followed by delayed definitive open reduction and internal fixation has been widely employed by United Kingdom (UK) and US maxillofacial teams. Published reports from these settings have generally shown acceptable outcomes with relatively low complication rates; however, most available studies consist of small case series or case reports, often involving military personnel treated in highly specialized medical facilities [19,20,21,22].

The management of war-related mandibular injuries becomes even more challenging in austere environments such as eastern DRC. Delays in evacuation, limited access to specialized surgical expertise, and scarcity of dedicated maxillofacial fixation systems may significantly increase morbidity when treatment is delayed or suboptimal [8]. Surgeons in this region—often general surgeons or maxillofacial surgeons working with limited resources—are frequently required to manage complex mandibular injuries caused by high-velocity firearm or blast trauma. Sophisticated mandibular EF systems are typically unavailable, necessitating adaptation of treatment strategies to the realities of warfare, local infrastructure, and available medical resources [21]. In this context, small orthopedic external fixators may represent a pragmatic and accessible alternative.

The purpose of this study was therefore to evaluate the outcomes and complications of external fixation used as a primary and definitive treatment for complex war-related mandibular fractures in a resource-limited setting. Can small orthopedic external fixators provide effective fracture stabilization and acceptable clinical outcomes when used as definitive treatment for complex war-related mandibular fractures in an austere civilian setting? We hypothesized that, despite delayed presentation, severe comminution, and high contamination rates, the use of small orthopedic external fixators as definitive fixation would result in satisfactory bone healing with an acceptable complication rate in patients with war-related mandibular fractures treated in eastern DRC.

## 2. Materials and Methods

### 2.1. Study Design and Patient Selection

A retrospective observational study was conducted at the Oral, Maxillofacial and Reconstructive Surgery Unit of the Hôpital Provincial Général de Référence de Bukavu (HPGRB), Democratic Republic of Congo (DRC). This retrospective observational study was conducted in accordance with the STROBE guidelines for observational studies. All consecutive patients who sustained war-related mandibular injuries and were treated with external fixation between January 2017 and December 2024 were reviewed. HPGRB is a major regional trauma referral center with extensive experience in the management of war-related maxillofacial injuries. The hospital receives and treats the majority of civilian and military patients evacuated from combat zones by the International Committee of the Red Cross (ICRC) during the ongoing armed conflict in eastern DRC. For the purpose of this study, war-related injuries were defined as mandibular injuries caused by gunshots, blast mechanisms, missiles, grenades, or other explosive devices.

All patients were routinely scheduled for postoperative clinical follow-up at 6 months. 8 Patients who were unable to attend follow-up visits up to 6 months postoperatively were excluded from the analysis. A total of 91 patients met the inclusion criteria and were included in the final study cohort.

This study was approved by the Institutional Ethics Committee of the Université Catholique de Bukavu (approval number UCB/CIES/PB/032/2025). Written informed consent was obtained from all patients whose clinical photographs or radiological images were used for publication. The study was conducted in accordance with the principles of the Declaration of Helsinki.

### 2.2. Preoperative Assessment

**Figure 1:**
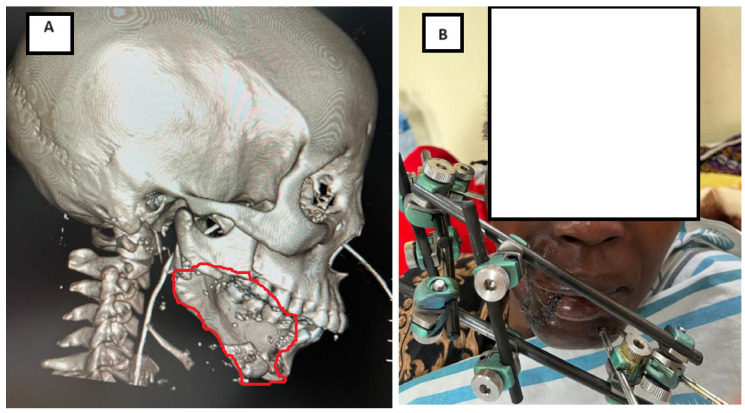
(**A**) Lateral view of three-dimensional computed tomography (CT) reconstruction showing the initial mandibular fracture with a segmental bone defect of the right mandibular body measuring approximately 5 cm. Defect is contoured in red. (**B**) Frontal clinical view of the patient with the external fixator in place (Orthofix Galaxy external fixation system).

All patients underwent a thorough clinical examination upon admission. Radiological assessment included anteroposterior and lateral oblique mandibular radiographs and/or computed tomography (CT) scans with three-dimensional reconstruction, when available, to characterize fracture patterns (Figure 1).

Data collected included patient demographics, mechanism of injury, fracture location, and presence of clinical infection at admission. Mandibular fracture patterns were classified as linear fractures, comminuted fractures, or fractures associated with bone loss resulting in continuity defects. The number of mandibular sites involved (single or multiple) was recorded. Additional data included the presence of oromucosal wounds, associated soft-tissue injuries, dental status, and associated injuries in other body regions. Intraoral contamination was defined as the presence of an open fracture communicating with the oral cavity, associated with saliva exposure, dental debris, or mucosal disruption. The time interval between injury and surgical fixation was documented. Adjunctive treatments, including tracheostomy, nasogastric feeding tube placement, or gastrostomy, were also recorded.

### 2.3. Surgical Procedure

All patients were managed according to the institutional protocol for war-related injuries. This protocol included tetanus prophylaxis, administration of broad-spectrum intravenous antibiotics (cefazolin 1 g three times daily, gentamicin 240 mg once daily, and metronidazole 500 mg intravenously three times daily), and extensive surgical debridement of all nonviable tissues. Antibiotic therapy was adapted according to clinical evolution. Thorough irrigation with high-pressure normal saline solution was systematically performed. Free-floating bone fragments, devitalized soft tissue, and retained foreign bodies were removed, while periosteum-bearing bone fragments were preserved whenever possible. Antibiotic therapy was continued for 3 days postoperatively and was adapted according to clinical evolution.

All procedures were performed under general anesthesia with nasal endotracheal intubation and placement of a throat pack, allowing free manipulation of the mandible for occlusal assessment. Tracheostomy was performed when clinically indicated.

Fracture reduction was achieved manually, guided by residual dentition when present, to restore appropriate occlusion. Stabilization was achieved using small orthopedic external fixators. Short cutaneous stab incisions were made at the pin insertion sites. Two threaded Steinmann pins (4.0 mm diameter) were inserted bicortically on each side of the fracture using a T-handle manual pin driver. Carbon rods were then connected to the pins and secured using appropriate clamps to achieve stable fixation. Sterile gauze dressings were placed around the pins during the early healing phase and maintained for the first 7 days.

Soft-tissue injuries were primarily repaired by direct suturing when contamination was minimal. In cases of significant contamination, wounds were managed with daily dressings and allowed to heal by secondary intention. When soft-tissue loss was extensive, local rotation-advancement flaps or pedicled flaps were used for coverage. The choice of pedicled flap was based on wound location, defect size, and patient characteristics.

Mandibular bone defects were subsequently reconstructed using corticocancellous bone grafts harvested from the iliac crest through an extraoral approach.

### 2.4. Postoperative Management and Follow-Up

Postoperatively, patients remained hospitalized for several weeks. External fixators were maintained in situ for a minimum of 6 weeks, and patients were placed on a soft diet during the fixation period. Pin-site care consisted of cleaning with sterile saline solution every other day. Because mouth opening was preserved during treatment, patients were instructed on appropriate oral hygiene measures and facial wound care. Chlorhexidine mouth rinses were prescribed.

Data collected during follow-up included duration of external fixation, need for bone grafting, soft-tissue reconstruction, and number of reoperations for infection. Treatment outcomes and factors associated with complications were analyzed.

Primary outcome measures included fracture healing and postoperative complications. Complications were defined as pin-tract infection, fracture-site infection, pin loosening, salivary leakage, limitation of mouth opening, and temporomandibular joint stiffness. Pin-tract infection was defined as the presence of erythema, discharge, pin loosening, or pin-tract osteomyelitis requiring curettage [23].

Clinical union was defined as the absence of pain or mobility at the fracture site on clinical examination and the absence of visible fracture lines on radiographic assessment at the time of external fixator removal. External fixators were removed under sedation. After removal, fixation components were cleaned and returned to the operating room inventory for reuse. In patients without initial bone defects, a healing period of 2 months or less was considered normal. Delayed union was defined as delayed fracture healing beyond 2 months requiring re-exploration and additional fixation, with or without bone grafting. Nonunion was defined as the absence of fracture healing beyond 6 months [23,24]. Fracture-related infection was diagnosed when purulent discharge from the fracture site necessitated surgical drainage and exploration [25].

### 2.5. Statistical Analysis

Statistical analysis was performed using SPSS software (version 31.0; IBM Corp., Armonk, NY, USA). Descriptive statistics were used to summarize the data. Continuous variables were expressed as mean ± standard deviation, while categorical variables were reported as frequencies and percentages.

Associations between clinical, radiological, and treatment-related variables and study outcomes (delayed union and fracture-related infection) were first assessed using the chi-square test or Fisher’s exact test, as appropriate. Binary variables were analyzed using odds ratios with 95% confidence intervals, while non-binary categorical variables were evaluated using chi-square or Fisher’s exact tests only. For each potential risk factor, odds ratios (ORs) with 95% confidence intervals (95% CI) were calculated to estimate the strength of association. A two-sided *p*-value < 0.05 was considered statistically significant.

## 3. Results

### 3.1. Patient Demographics

Ninety-one patients with war-related mandibular fractures treated with external fixation were included in the study. The mean age was 30.7 ± 11.6 years (range, 3–79 years), with the majority of patients aged between 21 and 40 years. Most patients were male (91.2%), and civilians accounted for 71.4% of the cohort, while 28.6% were military personnel.

High-velocity gunshot wounds were the predominant mechanism of injury, accounting for 94.5% of cases, whereas blast-related injuries from bombs or grenades were observed in 5.5%. Airway protection was frequently required: 30.8% of patients underwent tracheostomy, 61.5% required nasogastric feeding, and 3.3% required gastrostomy. Detailed demographic data are summarized in Table 1.

### 3.2. Mandibular Injury Characteristics

At admission, clinical evidence of wound infection was present in 27 patients (29.7%). The mean delay between injury and external fixation was 9.2 ± 6.6 days (range, 1–25 days); only 7.6% of patients were operated on within 48 h, whereas nearly half underwent surgery more than 7 days after injury.

Most fractures were severely comminuted (81.4%), and 16.5% were associated with segmental bone loss resulting in mandibular continuity defects. Fractures most frequently involved the mandibular body (58.2%) and angle (28.6%). Single-site mandibular involvement was observed in 76.9% of patients, while 23.1% sustained fractures involving two or more anatomical sites. Associated injuries in other body regions were present in 27.5% of patients, most commonly affecting the upper or lower limbs. Injury characteristics are detailed in Table 2.

### 3.3. Treatment Characteristics and Fracture Healing

**Figure 2:**
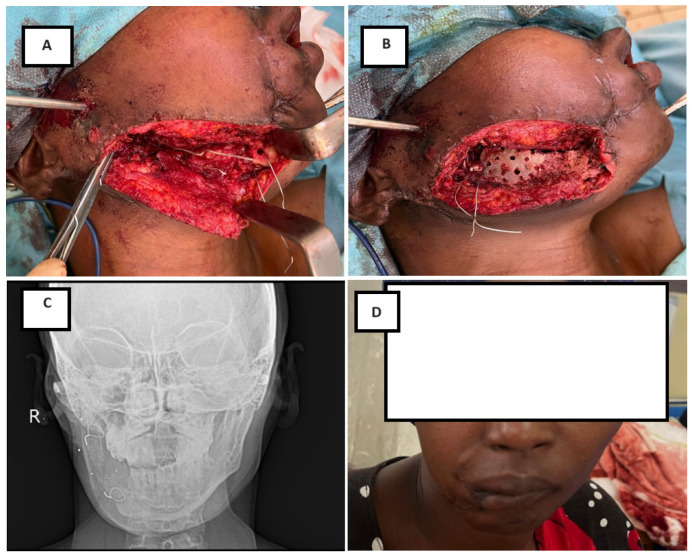
Secondary reconstruction following initial external fixation in the patient presented in Figure 1. (**A**) Intraoperative view through an extended submandibular approach allowing exposure of the defect and preparation of the recipient site. (**B**) Corticocancellous iliac crest bone graft shaped to fit the defect and fixed to the mandibular segments using wire fixation. (**C**) Six-month postoperative radiographic follow-up demonstrating restoration of mandibular continuity. (**D**) Six-month postoperative clinical appearance.

The external fixator was maintained for a mean duration of 8.8 ± 4.7 weeks (range, 3.1–26.4 weeks). Successful bone healing without secondary bone procedures was achieved in 71 patients (78.0%), with a mean healing time of 7.6 ± 3.0 weeks. Among these patients, 51 (72.5%) demonstrated normal healing, whereas 20 (27.5%) experienced delayed union. Figure 2 illustrates a representative case of a segmental mandibular defect managed with external fixation followed by delayed iliac crest bone grafting, resulting in restoration of mandibular continuity at 6-month follow-up.

Bone grafting was required in 20 patients (22.0%), including 19 iliac crest corticocancellous grafts and one rib graft. The mean interval between initial fixation and bone grafting was 77 ± 30 days (range, 33–124 days). Notably, only half of these patients had bone loss at initial presentation; the remaining patients developed secondary bone defects following serial debridement. Nonunion occurred in three patients (6.6%).

**Figure 3:**
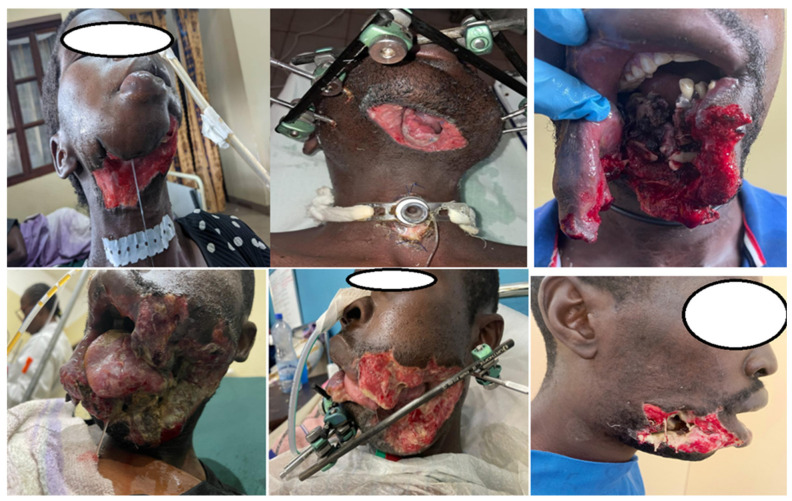
Clinical and radiographic aspects of a high-velocity gunshot injury causing a severely comminuted mandibular fracture with associated soft-tissue destruction and clinical evidence of infection at presentation.

Soft-tissue reconstruction was necessary in 31 patients (35.5%), most commonly using pedicled latissimus dorsi or pectoralis major flaps. Figure 3 illustrates the severity of soft-tissue and bony destruction typically observed in high-velocity war-related mandibular gunshot injuries included in this series. Small orthopedic external fixators were used in all cases, including Orthofix Galaxy devices in 79.1% and Hoffmann II fixators in 20.9%. Treatment characteristics and outcomes are summarized in Table 3.

**Figure 4:**
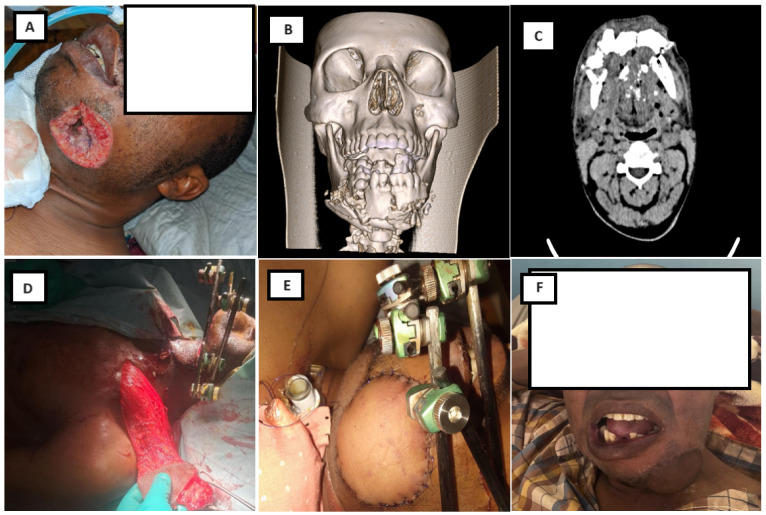
(**A**) Preoperative clinical photograph of a 45-year-old soldier who sustained a high-velocity gunshot injury to the face at close range, with extensive lacerating wounds involving the lower chin. (**B**,**C**) Preoperative computed tomography (CT) scan demonstrating complex comminuted mandibular fractures involving the left parasymphysis and the right angle. (**D**,**E**) Intraoperative views showing soft-tissue reconstruction using a pedicled latissimus dorsi flap and stabilization of the mandibular fractures with a small orthopedic external fixator (Orthofix Galaxy). (**F**) Clinical appearance after removal of the external fixator at 6 weeks, demonstrating satisfactory facial contour and an interincisal mouth opening greater than 35 mm. Published with the patient’s informed consent.

Univariate analysis identified intraoral contamination, presence of bone loss, need for bone grafting, gastrostomy, and a higher number of surgical procedures as factors significantly associated with delayed union (Table 4). Figure 4 shows a representative case highlighting functional and aesthetic outcomes following external fixation and soft-tissue reconstruction, including satisfactory facial contour and preserved mouth opening.

### 3.4. Complications

**Figure 5:**
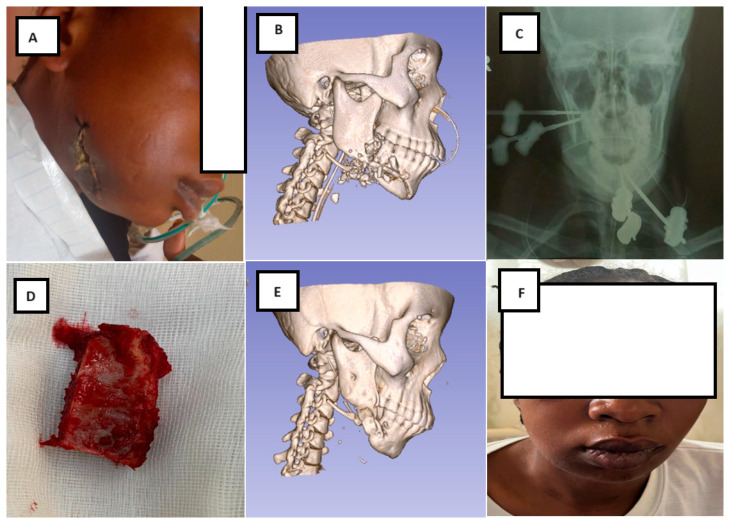
(**A**–**C**) A 36-year-old female patient with a high-velocity gunshot injury to the mandible, presenting with an extensive comminuted fracture and clinical evidence of wound infection at admission, treated with external fixation. (**C**) Postoperative radiograph showing pin placement, adequate fracture reduction, and a secondary bone defect following serial surgical debridement. (**D**) Corticocancellous iliac crest bone graft prepared for mandibular reconstruction. (**E**) Computed tomography (CT) scan obtained 6 months postoperatively demonstrating fracture healing and restoration of mandibular continuity. (**F**) Final clinical appearance at 6-month follow-up.

Overall, 33 patients (36.3%) experienced at least one complication (Table 5). Fracture-site infection was the most frequent complication, occurring in 30.8% of patients, followed by pin loosening, pin-tract infection, osteomyelitis with sequestration, and salivary leakage. Figure 5 presents a representative case of a high-velocity mandibular gunshot injury with clinical evidence of infection at admission, managed by external fixation, serial debridement, and delayed bone reconstruction.

Factors significantly associated with fracture-site infection included clinical evidence of wound infection at admission, presence of bone loss, requirement for bone grafting, and an increased number of surgical procedures (Table 6).

## 4. Discussion

This study demonstrates that small orthopedic external fixators can be used as a primary and definitive treatment for complex war-related mandibular fractures in a resource-limited setting, achieving acceptable bone union and functional outcomes despite severe injury patterns, high contamination rates, and delayed presentation. External fixation provided stable fracture immobilization, preserved soft-tissue viability, and allowed staged reconstruction when needed, without reliance on internal fixation systems that are often unavailable or contraindicated in this context.

### 4.1. Patient Demographics and Injury Context

In this series, most patients were young adult males, with the highest incidence observed in the 20–29-year age group. This demographic profile is consistent with previous reports from conflict zones, including Iraq and Afghanistan, where young men constitute the population most exposed to direct combat-related injuries [26]. A substantial proportion of patients received initial care in facilities located in or near combat zones before being transferred to our center, often with limited surgical resources.

As expected in this setting, many patients presented late with contaminated or clinically infected wounds, reflecting prolonged evacuation times and the absence of structured trauma systems. Unlike reports from Iraq, Afghanistan, and Israel—where blast injuries from bombs, grenades, or antipersonnel mines predominate [27,28]—the overwhelming majority of mandibular injuries in our cohort were caused by high-velocity rifle bullets (94.5%). Zachar et al., analyzing U.S. military casualties between 2001 and 2011, similarly reported explosives as the dominant mechanism of injury (61.3%), with ballistic trauma accounting for only 12.5% [28]. This contrast highlights important regional differences in weaponry and injury mechanisms.

Tracheostomy was frequently required, mainly due to extensive combined soft- and hard-tissue injuries and the anticipated need for multiple surgical procedures. In this context, tracheostomy remains preferable to repeated endotracheal intubation, as also reported in other military and civilian war-injury series [21,28,29].

### 4.2. Fracture Patterns and Rationale for External Fixation

Mandibular fractures most commonly involved the body and angle, consistent with previous studies of battle-related maxillofacial injuries [26,27,28]. The high proportion of comminuted fractures (82.5%) reflects the extensive energy transfer associated with rifle bullets, which are known to cause severe fragmentation, bone loss, and soft-tissue avulsion [30].

External fixation is particularly suited to these injury patterns, where bone fragments are often devascularized, contamination from the oral cavity is frequent, and dentition is insufficient to permit stable maxillomandibular fixation [26,31]. By minimizing periosteal stripping and avoiding the implantation of internal hardware in contaminated tissues, external fixation may reduce the risks of infection, osteomyelitis, and nonunion. In contrast, open reduction and internal fixation (ORIF) in severely comminuted ballistic injuries has been associated with high rates of wound dehiscence and infection, particularly in the acute phase [32]. Consequently, U.S. and U.K. maxillofacial teams have traditionally avoided ORIF during forward deployment in Iraq and Afghanistan, except in carefully selected cases [27].

### 4.3. Treatment Strategy and Outcomes

The overall bone union rate in this study was 78%, despite delayed treatment, frequent infection at admission, and a high prevalence of bone loss. Direct comparison with the literature is challenging, as union rates are inconsistently reported in studies focusing on war-related mandibular injuries [19,26]. In our cohort, delayed union was significantly associated with bone loss, the need for bone grafting, intraoral contamination, and a higher number of surgical procedures, reflecting the cumulative impact of injury severity and repeated debridement.

External fixation served as definitive stabilization throughout the treatment course. Internal fixation was not used before or after bone grafting, primarily due to limited availability of plates and concerns regarding infection. Bone grafts were secured using wire fixation, and external fixators were maintained until clinical and radiographic evidence of healing was observed. Although conversion from external to internal fixation has been advocated in other settings [21], this approach was not feasible in our context.

Corticocancellous iliac crest grafts were used to reconstruct mandibular continuity defects, which were generally smaller than 6 cm. No vascularized bone transfers were performed. Consistent with previous recommendations, bone grafting was delayed until soft-tissue conditions had stabilized, as immediate grafting in contaminated ballistic wounds is associated with high failure rates [4,17,27,33].

The mean duration of external fixation in our study (61.5 ± 32.8 days) was slightly shorter than the 88.6 days reported by Rose et al. in a series of mandibular gunshot fractures treated with external fixation [29].

Small orthopedic external fixators were selected because they were readily available and technically adaptable. Although devices such as the Hoffmann II fixator have been successfully used in mandibular and midface fractures [34,35], orthopedic fixators remain bulky and poorly contoured for facial anatomy [16,33]. Dedicated mandibular external fixators exist but are prohibitively expensive in low-income settings [36,37]. This underscores the need for affordable, locally adapted fixation systems in developing countries [38].

### 4.4. Complications

The overall complication rate was 36.3%, comparable to the 35.2% reported by Ellis et al. for comminuted gunshot mandibular fractures treated with external fixation [36]. While this rate may appear high, it must be interpreted in light of the extreme severity and contamination of war-related injuries. Complication rates reported for ORIF and maxillomandibular fixation in similar contexts range from 11% to 18% [17,36], but these techniques are often applied in less severe cases.

Fracture-site infection was the most common complication. The infection rate in our series exceeded that reported by Rose et al. (14%) [29] but was strongly associated with clinical infection at admission, bone loss, and the need for bone grafting. Delayed treatment and prolonged contamination—common in low-income countries with limited evacuation systems—likely contributed to this increased risk [23,26,39].

Pin-tract infections were relatively uncommon (5.6%) compared with orthopedic series reporting rates between 27.3% and 59.1% [40]. Most were superficial and resolved with local care and antibiotics; only two cases required curettage for pin-tract osteomyelitis.

Pin-tract infection remains a known limitation of external fixation. It is primarily driven by bacterial colonization along the pin–bone interface, local soft tissue motion, and impaired host immunity. Recent experimental and clinical studies have explored antibiotic-coated pins and local drug-releasing biomaterials to reduce bacterial adherence and biofilm [41,42,43,44,45,46]. However, despite the growing interest in antibacterial and drug-releasing biomaterials, most of these strategies remain experimental, and their clinical translation is limited. Anti-adhesive surface modifications, in particular, may impair osseointegration and are therefore not recommended for load-bearing bone implants [47]. Targeted local drug delivery appears more promising, but its effectiveness depends on complex local pharmacokinetics, including biological fluid inactivation, microbiome interactions, and tissue characteristics, which remain difficult to control in clinical settings. Beyond infection control, emerging drug-releasing biomaterials may also actively enhance tissue and bone healing. In particular, strontium-releasing biomaterials have demonstrated osteogenic and angiogenic properties, promoting bone formation while simultaneously modulating osteoclastic activity, which may contribute to improved bone repair and soft-tissue integration in complex and contaminated defects [48]. Although such technologies are not currently available in our setting, they represent promising adjuncts that could significantly reduce infection rates in austere environments.

Salivary fistulas resulting from placement of pins are uncommon. When they do occur, resolution occurs spontaneously during fixation some times in the two weeks [49]. We simply had to reassure the patient. The major problem is that this complication can be mistaken for discharge, which can mislead our approach and lead us to over-treat an infection that is not actually present.

Although no refracture was observed in our series, the restoration and maintenance of a stable occlusion is recognized as a critical factor for successful mandibular fracture healing, and occlusal derangement or persistent excessive loading may theoretically contribute to delayed union or malunion in the absence of adequate protection and reinforcement. Previous literature emphasizes the importance of achieving and maintaining proper occlusal relationships and mechanical stability to minimize complications after mandibular fracture repair [25,50,51].

### 4.5. Strengths and Limitations

This study has several limitations inherent to its retrospective design. Functional outcomes such as occlusion quality, temporomandibular joint mobility, and patient-reported quality of life could not be systematically assessed. Additionally, the lack of individual-level data precluded multivariate analysis, limiting conclusions to associations rather than independent predictors. Systemic comorbidities such as diabetes mellitus could not be systematically assessed due to the emergency context and incomplete medical histories, which may have influenced infection risk. The association between gastrostomy and delayed union should be interpreted cautiously due to the small number of affected patients.

Nevertheless, to our knowledge, this represents the largest reported civilian cohort in which external fixation was used as the sole definitive treatment for complex war-related mandibular fractures. The study provides valuable insight into the management of high-energy ballistic mandibular injuries in a high-risk, resource-limited environment, where conventional fixation systems are unavailable.

## 5. Conclusions

Patients treated with external fixation in this series sustained severe mandibular injuries characterized by extensive comminution, soft-tissue damage, infection, and delayed presentation. Although external fixation is not routinely used for mandibular fractures in civilian practice, this study demonstrates that small orthopedic external fixators can provide satisfactory bone union and acceptable complication rates when applied to severe high-velocity ballistic injuries in low-resource settings. External fixation should therefore be considered as a valid and pragmatic treatment option for complex mandibular war injuries where dedicated maxillofacial fixation systems are unavailable.

## Figures and Tables

**Table 1:** Sociodemographic characteristics of patients treated with external fixation.

Characteristic	*N* = 91
Age (years), mean ± SD	30.7 ± 11.6
Age group, *n* (%)	
<20 years	4 (4.4)
20–49 years	79 (86.8)
≥50 years	8 (8.8)
Sex, *n* (%)	
Male	83 (91.2)
Female	8 (8.8)
Patient status, *n* (%)	
Civilian	65 (71.4)
Military	26 (28.6)
Initial treatment before transfer, *n* (%)	
Surgical debridement	30 (33.0)
No prior treatment	61 (67.0)
Airway and nutritional support at admission, *n* (%)	
Tracheostomy	28 (30.8)
Gastrostomy	3 (3.3)
Nasogastric feeding tube	56 (61.5)

**Table 2:** Mandibular injury characteristics of patients treated with external fixation.

Characteristic	*N* = 91
Mechanism of injury, *n* (%)	
High-velocity gunshot	86 (94.5)
Blast injury (bomb, grenade, other explosives)	5 (5.5)
Oromucosal wound, *n* (%)	
Yes	76 (83.5)
No	15 (16.5)
Clinical evidence of wound infection at admission, *n* (%)	
Yes	27 (29.7)
No	64 (70.3)
Fracture pattern, *n* (%)	
Linear fracture	1 (1.1)
Comminuted fracture	75 (82.4)
Segmental bone loss (continuity defect)	15 (16.5)
Primary fracture location *, *n* (%)	
Symphysis	4 (4.4)
Body	53 (58.2)
Angle	26 (28.6)
Ramus	8 (8.8)
Number of mandibular sites involved, *n* (%)	
Single site	70 (76.9)
Two sites	20 (22.0)
≥Three sites	1 (1.1)
Side affected, *n* (%)	
Left	46 (50.5)
Right	42 (46.2)
Midline (symphysis)	3 (3.3)
Dental status, *n* (%)	
Intact dentition	18 (19.8)
Local tooth mobility and loss at fracture site	35 (38.5)
Tooth loss involving one hemi-arch	11 (12.1)
Tooth loss involving one full arch	13 (14.3)
Extensive tooth loss (≥two arches)	9 (9.9)
Associated maxillary dentoalveolar injury	3 (3.3)
Associated craniofacial fractures, *n* (%)	
Yes	27 (29.7)
No	64 (70.3)
Associated injuries in other body regions, *n* (%)	
Upper or lower limb injuries	19 (20.9)
Thoracic and/or abdominal injuries	6 (6.6)
None	66 (72.5)
Time from injury to external fixation, *n* (%)	
<2 days	7 (7.6)
3–7 days	42 (46.2)
>7 days	42 (46.2)

* For patients with multiple fracture sites, the most clinically significant location was recorded.

**Table 3:** Treatment characteristics and outcomes of patients treated with external fixation.

Characteristic	*N* = 91
Duration of external fixation, mean ± SD (range)	61.5 ± 32.8 days (22–185)
Fracture healing outcome in patients without initial bone defect, *n* (%)	
Normal union	51 (72.5)
Delayed union	20 (27.5)
Nonunion	0
Soft-tissue reconstruction with pedicled flap, *n* (%)	
Yes	31 (34.1)
Latissimus dorsi flap	19
Pectoralis major flap	10
Submental flap	2
No	60 (65.9)
Bone grafting required, *n* (%)	
No	71 (78.0)
Iliac crest graft	19 (20.9)
Rib graft	1 (1.1)
Combined soft-tissue and bony defect, *n* (%)	
Yes	13 (14.3)
No	78 (85.7)
Number of surgical procedures, mean ± SD (range)	3.1 ± 2.2 (1–10)
Number of surgical procedures, *n* (%)	
≤2	46 (50.5)
3–4	28 (30.8)
≥5	17 (18.7)
Type of orthopedic external fixator used, *n* (%)	
Hoffmann II	72 (79.1)
Orthofix Galaxy (small)	19 (20.9)
Time intervals, mean ± SD (range)	
Injury to external fixation	9.2 ± 6.6 days (1–25)
External fixation to bone grafting	77.3 ± 30.5 days (33–124)
External fixation to soft-tissue coverage	5.3 ± 6.9 days (0–23)
External fixation to pin-tract infection	38.0 ± 31.2 days (11–92)

**Table 4:** (A) Factors associated with delayed union after external fixation for war-related mandibular fractures. (B) Non-binary factors evaluated for association with delayed union *.

**(A)**				
**Factor**	**Delayed Union (*n* = 34)**	**Normal Union (*n* = 57)**	Odds Ratio (95% CI) *	***p* Value**
Intraoral contamination				
Yes	25	51	3.1 (1.1–8.4)	0.047
No	9	6	Reference	
Bone loss at presentation				
Yes	10	5	4.3 (1.3–14.3)	0.010
No	24	52	Reference	
Bone grafting required				
Yes	13	7	4.4 (1.5–13.0)	0.004
No	21	50	Reference	
Gastrostomy required				
Yes	2	1	3.5 (0.3–38.0)	0.011
No	32	56	Reference	
Soft-tissue reconstruction required				
Yes	13	18	1.3 (0.5–3.4)	0.510
No	21	39	Reference	
Tracheostomy required				
Yes	14	20	2.2 (0.9–5.3)	0.090
No	20	37	Reference	
Associated craniofacial injuries				
Yes	8	19	0.6 (0.2–1.6)	0.322
No	26	38	Reference	
Associated injuries in other body regions				
Yes	11	14	1.5 (0.6–3.8)	0.421
No	23	43	Reference	
Debridement before transfer				
Yes	11	20	0.9 (0.3–2.2)	0.790
No	23	37	Reference	
**(B)**				
**Factor**	**Delayed Union (** ***n*** ** = 34)**		**Normal Union (** ***n*** ** = 57)**	***p*** ** Value**
Age group (years)				0.627
<20	2		6	
20–49	29		48	
≥50	3		3	
Mechanism of injury				0.268
High-velocity gunshot	28		55	
Blast injury	2		3	
Time from injury to external fixation				0.928
<2 days	3		4	
3–5 days	15		27	
6–10 days	16		26	
Fracture location				0.336
Body	13		38	
Angle	12		12	
Symphysis	7		2	
Ramus	1		1	
Number of fracture sites				0.336
1 site	27		43	
2 sites	6		14	
≥3 sites	1		0	
Extent of dentoalveolar injury				0.910
None	8		10	
Isolated	16		30	
Extensive	5		8	
With maxillary involvement	5		9	

(A) * Odds ratios with 95% confidence intervals were calculated for dichotomous variables only. Variables with more than two categories were analyzed using chi-square or Fisher’s exact tests without estimation of effect size to avoid arbitrary categorization. (B) * Comparisons were performed using chi-square or Fisher’s exact tests, as appropriate.

**Table 5:** Postoperative complications after external fixation.

Complication *	Patients, *n* (%)
At least one complication	33 (36.3)
Fracture-site infection	28 (30.8)
Pin-tract infection	6 (5.6)
Pin loosening	13 (14.3)
Salivary leakage	1 (1.1)

* Percentages are calculated based on the total number of patients (N = 91). Complications were not mutually exclusive, and some patients experienced more than one complication.

**Table 6:** (A) Binary factors associated with surgical site infection after external fixation Methodological quality of included cohort studies. (B) Non-binary factors evaluated for association with surgical site infection *.

(A)				
Factor	With Infection (*n* = 28)	Without Infection (*n* = 63)	Odds Ratio * (95% CI)	*p*-Value
Clinical wound infection at admission				
Yes	13	14	3.0 (1.2–7.7)	0.020
No	15	49	Reference	
Intraoral contamination				
Yes	26	50	3.4 (0.7–16.0)	0.093
No	2	13	Reference	
Bone loss at presentation				
Yes	8	7	3.2 (1.0–10.4)	0.042
No	20	56	Reference	
Bone grafting required				
Yes	10	10	2.9 (1.1–7.7)	0.036
No	18	53	Reference	
Soft-tissue reconstruction required				
Yes	11	20	1.7 (0.7–4.2)	0.484
No	17	43	Reference	
Debridment before transfer				
Yes	10	20	1.2 (0.5–3.1)	0.710
No	18	43	Reference	
Associated craniofacial injuries				
Yes	7	20	0.7 (0.3–1.9)	0.517
No	21	43	Reference	
Associated injuries in other body regions				
Yes	10	15	1.8 (0.7–4.7)	0.243
No	18	48	Reference	
**(B)**				
**Factor**	**With Infection (*n* = 28)**		**Without Infection (*n* = 63)**	***p*** ** Value**
Mechanism of injury				0.490
High-velocity gunshot	26		60	
Blast injury (bomb, grenade, explosive)	2		3	
Time from injury to external fixation				0.887
<2 days	2		5	
3–7 days	12		30	
>7 days	14		28	
Fracture location				0.800
Body	13		38	
Angle	12		12	
Symphysis	7		2	
Ramus	1		1	
Number of mandibular fracture sites				0.638
1 site	23		47	
2 sites	5		15	
≥3 sites	0		1	
Extent of dentoalveolar injury				0.781
None	5		13	
Isolated	15		31	
Extensive	5		8	
With maxillary involvement	3		11	

(A) * Odds ratios (OR) with 95% confidence intervals (CIs) were calculated for dichotomous variables only. (B) * Comparisons were performed using chi-square or Fisher’s exact tests, as appropriate.

## Data Availability

The original contributions presented in this study are included in the article.

## Abbreviations

The following abbreviations are used in this manuscript:

DRC	Democratic Republic of the Congo.
ORIF	Open reduction and internal fixation.
MMF	Maxillomandibular fixation.
HPGRB	Hôpital Provincial Général de Référence de Bukavu.
UK	United Kingdom.
ICRC	International Committee of the Red Cross.
EF	External fixation.
